# microRNAs in aged sperm confer psychiatric symptoms to offspring through causing the dysfunction of estradiol signaling in early embryos

**DOI:** 10.1038/s41421-022-00414-1

**Published:** 2022-07-05

**Authors:** Gaoli Liang, Xiaoju Zhu, Zixuan Zhang, Li Chen, Huijin Feng, Shoubin Zhan, Huanhuan Hu, Ranran Yu, Chen-Yu Zhang, Zhichun Feng, Bing Yao, Yanbo Wang, Xi Chen

**Affiliations:** 1 Nanjing Drum Tower Hospital Center of Molecular Diagnostic and Therapy, State Key Laboratory of Pharmaceutical Biotechnology, Jiangsu Engineering Research Center for MicroRNA Biology and Biotechnology, NJU Advanced Institute of Life Sciences (NAILS), School of Life Sciences, Nanjing University, Nanjing, Jiangsu China; 2 Center of Reproductive Medicine, Jinling Hospital, Clinical School of Medical College, Nanjing University, Nanjing, Jiangsu China; 3 Research Unit of Extracellular RNA, Chinese Academy of Medical Sciences, Nanjing, Jiangsu China; 4 Pingshan Translational Medicine Center, Shenzhen Bay Laboratory, Shenzhen, Guangdong China; 5 Department of Pediatrics, the Seventh Medical Center of PLA General Hospital, Beijing, China; 6 Chemistry and Biomedicine Innovation Center (ChemBIC), Nanjing University, Nanjing, Jiangsu China

**Keywords:** miRNAs, Mechanisms of disease

Dear Editor,

In recent decades, delayed child-bearing has become an increasingly prevailing trend in most developed countries. This demographic shift has also led to concerns about the potential adverse effects of advanced parental age on the health of the offspring. Indeed, advanced paternal age has been frequently linked to a number of negative psychiatric outcomes in offspring, including autism, schizophrenia, and bipolar disorder, as well as intellectual and academic problems, but the underlying molecular mechanism remains enigmatic^1^. Recently, sperm small RNAs (sRNAs) have been identified as a causal vector that contributes to the intergenerational inheritance of paternal phenotypes (e.g., mental stress and metabolic disorders) in mammals^2,3^. In essence, sperm are capable of shuttling a diverse set of sRNAs into the oocyte as part of fertilization, and inherited sRNAs utilize different mechanisms to modulate gene regulatory networks during early embryonic development and even induce long-term behavioral and physiological changes in adult offspring^2,3^. However, whether sperm sRNAs are sensitively influenced by advanced age and contribute to the inheritance of psychiatric disorders in offspring is poorly understood.

F0-aged males (12–14 months, F0-aged) and F0 young males (8 weeks, F0-Ctl) were separately mated with young females (8 weeks), and their offspring (F1-aged vs. F1-Ctl) were screened for behavioral abnormalities (e.g., anxiety, social dysfunction, and depression) after becoming adults (Supplementary Fig. S1a). Under baseline conditions, F1-aged and F1-Ctl traveled an equal total distance in the open-field tests and spent an equal time in the center area (Supplementary Fig. S1b, c). However, after exposure to an acute restrained stress (ARS) lasting for 30 min, F1-aged tended to have less residence time in the center area of the open-field arena (Supplementary Fig. S2a–c). In elevated plus maze tests, F1-aged and F1-Ctl had similar open arm entry times and spent similar amount of time in open arms under basal conditions (Supplementary Fig. S1d, e). However, after ARS exposure, F1-aged spent significantly less time in the open arms compared with F1-Ctl (Supplementary Fig. S2d, e). These results suggest that the offspring of aged male mice are more susceptible to anxiety in response to adverse stimuli. In the first session of three-chamber social tests to assess sociability, both F1-aged and F1-Ctl displayed normal behaviors at baseline and preferred to stay in the chamber with a stranger mouse 1 and to sniff the stranger mouse 1 over an empty cage; in the second session to assess social novelty preference, both F1-aged and F1-Ctl spent more time sniffing the stranger mouse 2 (Supplementary Fig. S1f, g). However, after stimulating with ARS, while F1-Ctl did exhibit a preference for a novel mouse in either the sociability or social novelty preference portion of the test, F1-aged spent nearly equal amounts of time sniffing strangers 1 and 2 in the social novelty preference test (Supplementary Fig. S2f, g), suggesting that the offspring of aged male mice are hypersensitive to deficits in social recognition memory when exposure to adverse effects. Compared to F1-Ctl, F1-aged did not show depression-like behaviors in the forced swimming test and sucrose preference test (Supplementary Fig. S1h, S2h). Overall, the offspring born to aged male mice are more vulnerable to psychiatric symptoms, characterized by increased anxiety levels and social communication defects.

To address whether sperm sRNAs would play a causal role in the intergenerational inheritance of age-induced psychiatric problems, we purified sRNAs (< 200 nt) from the sperm of F0-aged/F0-Ctl and injected them into normal zygotes. Then the embryos were transferred into surrogate mothers, and the corresponding offspring (sRNA-aged vs. sRNA-Ctl) were subjected to behavioral tests of anxiety and social interaction once they were adult (Supplementary Fig. S3a). In open-field test, sRNA-aged performed normally at baseline and had similar travel distance and central residence time compared with sRNA-Ctl (Supplementary Fig. S3b, c). However, when exposed to ARS, sRNA-aged acquired pronounced anxiety-like phenotypes, spending significantly less time in the center area of the open-field arena (Fig. 1a, b; Supplementary Fig. S4a, b). In elevated plus maze test, sRNA-aged tended to spend less time than sRNA-Ctl in the open arms, both under baseline conditions and after ARS exposure (Fig. 1c, d; Supplementary Figs. S3d, e, S4c). For the analyses of social behaviors, sRNA-Ctl exhibited a preference for a novel mouse in either the sociability or social novelty preference portion of the three-chamber social tests; in contrast, sRNA-aged spent significantly shorter time with strangers in the social novelty preference test, both under baseline conditions and after ARS exposure (Fig. 1e, f; Supplementary Fig. S3f, g, S4d). Thus, injection of sperm sRNA into zygotes produces same behavioral abnormalities and psychiatric symptoms in the resulting offspring as those born to aged father.

**Fig. 1:**
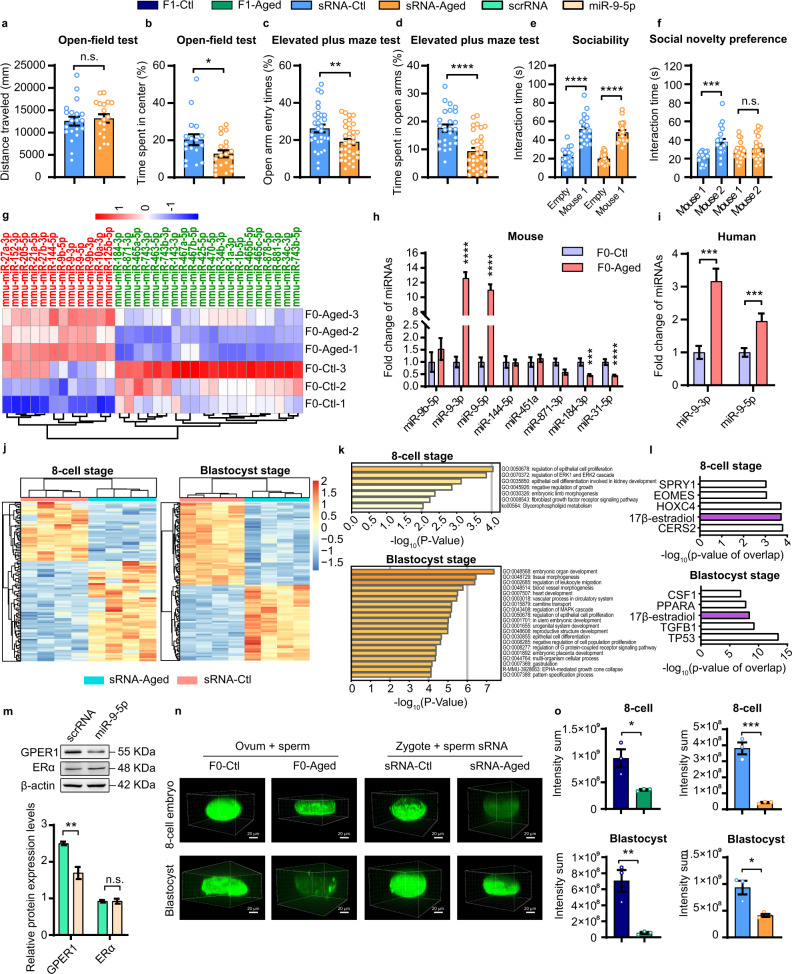
miRNAs in the sperm of aged mice confer anxiety and social dysfunction to the offspring through inappropriately regulating 17β-estradiol signaling in early embryos. **a**–**f** Behavioral paradigm in IVF offspring born to zygotes injected with sperm sRNA (sRNA-aged vs. sRNA-Ctl) after exposure to ARS: total distance traversed in the open arena (**a**), percent time spent in central areas of the arena (**b**) (*n* = 17–20 per group); percent number of entries into open arms (**c**), percent time spent in the open arms (**d**) (*n* = 25–26 per group); total time spent sniffing the stranger mouse 1 and empty (**e**), total time spent sniffing the stranger mouse 1 and mouse 2 (**f**) (*n* = 16–20 per group). **g** Heatmap comparison illustrating sperm miRNAs that were significantly upregulated (red) or downregulated (green) in F0-aged vs. F0-Ctl (*n* = 3 per group). **h** Quantitative RT-PCR analysis of the expression levels of miRNAs in the sperm derived from F0-aged and F0-Ctl (*n* = 8 per group). **i** Quantitative RT-PCR analysis of the expression levels of miRNAs in the sperm derived from aged and young human donors (*n* = 23 per group). **j** Heatmap comparison illustrating the top 100 DEGs between sRNA-aged and sRNA-Ctl at 8-cell stage (left) and blastocyst stage (right) (*n* = 4 per group). **k** GO analysis of the DEGs between the 8-cell stage (upper) and blastocyst stage (lower). **l** IPA tool identifying the functions and upstream regulators associated with DEGs at the 8-cell stage (upper) and blastocyst stage (lower). **m** Western blot analyses (upper) and densitometry quantification (lower) of GPER1 and ERα proteins in N2A cells transfected with scrambled RNA (scrRNA) or miR-9-5p (*n* = 4 per group). ERα harboring no binding sites for miR-9-5p served as a negative control. **n** Immunofluorescence analysis of GPER1 in the 8-cell embryos and blastocysts derived with IVF from F0-aged or F0-Ctl (left), or in the 8-cell embryos and blastocysts developing from zygotes injected with sperm sRNAs from F0-aged or F0-Ctl (right). **o** Quantification of the immunofluorescence staining intensity (*n* = 3 per group).

To identify which specific subtypes of sperm sRNAs cause abnormalities in offspring, we examined the sRNA profiles of sperm derived from F0-aged and F0-Ctl by sRNA deep sequencing. Length distribution analysis and sRNA annotation and classification revealed that rRNA-derived small RNA (rsRNA), microRNA (miRNA), Piwi-interacting RNA (piRNA), and tRNA-derived small RNA (tsRNA) were the most abundant sRNAs in sperm (Supplementary Fig. S5). We next focused on sperm miRNAs, because miRNAs, rather than other sRNA types, were abundantly expressed and upregulated in the sperm of F0-aged compared with F0-Ctl. Indeed, a number of miRNAs were significantly increased in the sperm of F0-Aged (Fig. 1g). Quantitative RT-PCR analysis confirmed that miR-9-3p and miR-9-5p were consistently increased in the sperm of F0-aged (Fig. 1h). Interestingly, miR-9-3p and miR-9-5p were validated to be significantly increased in the sperm derived from aged human donors (Fig. 1i).

To investigate the mechanism by which sperm sRNAs contribute to age-induced psychiatric symptoms, we injected sperm sRNAs from F0-aged or F0-Ctl into zygotes and assessed alteration of gene profiles by single-cell transcriptome RNA sequencing when the embryos developed to embryonic day 2.0 (E2.0, corresponding to 8-cell stage) and to embryonic day 3.5 (E3.5, corresponding to blastocyst stage). In both 8-cell embryos and blastocysts, a number of genes were significantly changed (fold-change > 2 and *P* < 0.05) in the groups injected with sperm sRNAs from F0-aged compared with those injected with sperm sRNAs from F0-Ctl (Fig. 1j). Gene ontology (GO) analysis of the differentially expressed genes (DEGs) identified enrichment of GO clusters whose primary functions are frequently linked to embryonic development and cell proliferation/differentiation (Fig. 1k). To uncover the signaling pathway and interaction network that cause large-scale abnormal gene expression, ingenuity pathway analysis (IPA) was performed to identify the functions and upstream regulators associated with DEGs. Beta-estradiol (17β-estradiol) pathway was identified as a common upstream regulator sharing between 8-cell embryos and blastocysts (Fig. 1l). When sperm sRNAs from F0-aged or F0-Ctl were injected into normal zygotes and the alteration of the genes downstream the 17β-estradiol signaling was assessed at 2-cell, 4-cell, 8-cell, blastocyst, and E12.5 stages, a gradual increase in the expression of these genes was observed starting from 8-cell stage, and a widespread dysregulation of these genes was observed at E12.5 stage (Supplementary Fig. S6).

17β-estradiol influences multiple aspects of placental function and exerts a central role in embryonic development, especially for neuronal development and plasticity^4^. 17β-estradiol elicits its biological effects through three types of estrogen receptor (ER): ERα, ERβ, and G protein-coupled oestrogen receptor 1 (GPER1)^4^. Intriguingly, GPER1 was predicted as a direct target gene of miR-9-5p (Supplementary Fig. S7a). As anticipated, miR-9-5p was experimentally validated to directly inhibit GPER1 protein but not mRNA in N2A cells (Fig. 1m; Supplementary Fig. S7b), consistent with a miRNA-mediated post-transcriptional regulatory mechanism. Since GPER1 is classically localized to plasma membrane, we performed immunofluorescent staining and visualized the location and expression of GPER1 by confocal microscopy. While green fluorescence was predominantly assembled in the membrane of 8-cell embryos and blastocysts, the fluorescence intensity of GPER1 in the 8-cell embryos and blastocysts derived with IVF from F0-aged was significantly lower than those from F0-Ctl (Fig. 1n, o). Likewise, in embryos developing from zygotes injected with sperm sRNAs from F0-aged, fluorescence intensity of GPER1 was also decreased at the 8-cell and blastocyst stages (Fig. 1n, o). However, *GPER1* mRNA levels were unchanged in the embryos of sRNA-aged at 2-cell, 4-cell, 8-cell, blastocyst, and E12.5 stages (Supplementary Fig. S7c). Moreover, when zygotes injected with miR-9-5p mimic were developing to 8-cell and blastocyst stages, GPER1 was again found to be decreased (Supplementary Fig. S7d, e). Consistently, when the zygotes injected with miR-9-5p or scrRNA were developing to adults and assessed by behavioral tests (miR-9-5p vs. scrRNA), miR-9-5p group performed normally in open-field test but exhibited significant anxiety-like behaviors after ARS stimulation in elevated plus maze tests, and displayed impaired social recognition ability both under baseline conditions and after ARS exposure in the social novelty preference portion of three-chamber social tests (Supplementary Fig. S8). Thus, injection of miR-9-5p into normal zygotes might at least partially recapitulate the psychiatric symptoms in offspring born to aged father. Finally, when sRNAs derived from the sperm of aged and young human donors were injected into normal zygotes, GPER1 has consistently reduced in 8-cell embryos and blastocysts of the groups injected with aged sperm sRNAs (Supplementary Fig. S7d, e). Meanwhile, when the blastocysts developed from the zygotes injected with human sperm sRNAs were compared with those injected with mouse sperm sRNAs, a substantial overlap of the significantly upregulated or downregulated genes was observed (Supplementary Fig. S9). Overall, inherited sRNAs from an aged father might inappropriately inhibit GPER1 during early embryonic development, thereby imprinting abnormalities in the estradiol signaling and causing a cascade change and profound downstream effects on the development of the nervous system, which eventually lead to a vulnerable phenotype for psychiatric problems in adult offspring.

## Supplementary information

Supplementary Figures and Tables
