# Supplementary material for: microRNAs in aged sperm confer psychiatric symptoms to offspring through causing the dysfunction of estradiol signaling in early embryos
Source: Cell Discov. 2022 Jul 5;8:63. doi: 10.1038/s41421-022-00414-1 (PMC9256735; doi:10.1038/s41421-022-00414-1)
Supplement: Supplementary file 1 — Supplementary Figures and Tables [file 41421_2022_414_MOESM1_ESM.pdf]

Supplementary information for

microRNAs in aged sperm confer psychiatric symptoms to offspring through causing the dysfunction of estradiol signaling in early embryos

(Gaoli Liang, *et al*)

Supplementary Methods

Mice

Inbred C57BL/6J strain and closed colony ICR were obtained from the GemPharmatech Co., Ltd. (Nanjing, China). Mice were reared according to a strict 12-hour alternate photoperiod (lighted on at 9:00 a.m. and lighted off at 9:00 p.m. every day) and were provided with sufficient food and water every day, unless otherwise specified. All animal care and handling procedures were performed in accordance with the National Institutes of Health Guide for the Care and Use of Laboratory Animals, and were approved by the Institutional Review Board of Nanjing University (Nanjing, China).

Breeding scheme: F1 offspring

Natural reproductive strategy of aged male mice

To get F1 offspring (F1-Aged and F1-Ctl), aged (12-14 months) and young (8-10 weeks) mice (F0 generation) were mated with female mice (6-8 weeks), respectively. The offspring of each group were screened for behavioral tests when they were adult (8-10 weeks).

Microinjection and embryo transfer strategies

The same batch of normal zygotes were randomly divided into two groups and injected with sRNA from the sperm of aged (12-14 months) or young (8-10 weeks) male mice, respectively. Sperm sRNA (concentration: 2 ng/μL) were microinjected into the cytoplasm of normal zygotes. The 2-cell stage embryos were transplanted into the ampulla of oviduct in the surrogate mice. The offspring born from the surrogate mice were called the sRNA-Aged group and sRNA-Ctl group, correspondingly.

Acute restrain stimulus (ARS)

For ARS, mice were confined for 30 minutes in a 50-ml cone-shaped tube with air outlets. Once the ARS has been performed, it was essential to let the mice rest for 30 minutes between the ARS and the behavioral test.

Behavioral tests

All behavior experiments were tested on adult mice of C57BL/6J background. On the day before the test, the mice would be taken to the test room and kept overnight. All experiments allowed to test only in the specific light cycle (09:00 to 18:00). Investigators were blinded to group assignment during behavioral experiments.

Open-field test

The open-field equipment was performed in an opaque hollow polypropylene box (25 cm × 25 cm × 50 cm) with lid. The square area (25 cm × 25 cm) at the bottom is the test area for spontaneous activities of mice. The camera on the lid of the box recorded the spontaneous movement of the test mice under dim light for 10 minutes. Clever TopScan software was used to perform further track and analysis. In the software analysis, the whole area was divided into nine equal squares (3 × 3), the middlemost square was defined as the central area and the 8 peripheral squares were defined as the rest area.

Elevated plus maze test

The maze was 50-cm high from the ground, arranged in a crisscross pattern with four arms. The four arms were divided into two open arms and two closed arms, with the same size (30 cm × 5 cm). The same arms were in opposite positions, only the closed arms were covered by a 20 cm high wall. At the beginning, the test mice were placed in the central area where the four arms crossed, facing an open arm. In the test space where there is no operator, accompanied by dim lights, the overhead camera recorded the 5-minute free exploration process of test mice. Data were analyzed using video-tracking software (TopScan; CleverSys Inc., Reston, VA), and the percentage of frequency and time in the open arms was calculated.

Three-chamber social interaction test

The equipment in three-chamber social interaction test included three same chambers (20 cm × 40 cm × 22 cm) and two demountable doors (3 cm × 5 cm). Test mice were first placed in the empty and interconnected chamber for 5-minute habituation, and then the same mice were placed in the center chamber for a two-session test. For the "sociability" session, metal cages were placed on one side of the chamber, respectively. One cage contained a strange male mouse (stranger I) and another was empty (empty). Both doors were opened and the test mice were placed in the middle chamber and recorded to explore freely for 10 minutes. In the "preference for social novelty" session, a new strange mouse (stranger II) was placed in the empty metal cage, and the stranger I was regarded as familiar mice. Then both doors were opened and the test mice were recorded to explore freely for another 10 minutes. The area of 2 cm around the metal cage was defined as the interaction area. The locomotor activity was recorded by a camera overhead. Clever TopScan Software was used to analyze the videos.

Forced swimming test

Forced swimming test has been used as a predictive model of depressive behaviors. Briefly, each mouse was placed in a vertical Plexiglas bucket (height = 25 cm, diameter = 18 cm), containing 15 cm water at 25° ± 1°C. Swimming activity was recorded for 6 minutes by a video camera. With the first minute considered acclimatization, only last 5 minutes of the test was analyzed by Clever TopScan Software.

Sucrose preference test

Sucrose preference test, a measure of anhedonia-like behaviors in mice, was assessed in a two-bottle choice test. Sucrose preference test needs to take 4 days. On the first day, the mice were individually housed to adapt environment. On the second day, the mice were provided with both 1% sucrose solution and plain water, and could drink freely. On the third day, the water and 1% sucrose bottles were switched, and the location of the water and 1% sucrose bottles was balanced across animals. After 3 days of habituation, the consumption of both water and sucrose were measured for 24 hours and expressed as (Δ weight sucrose) / (Δ weight sucrose + Δ weight water) × 100.

Sperm sample collection and sRNA sequencing

Mouse sperm isolation

Sperm samples from mice were extracted from the epididymal tail. To get mature sperm, the epididymal tail was placed in 5 mL phosphate-buffered saline (PBS) after being slightly cut into small pieces and then incubated at 37°C for 15 minutes. In order to eliminate the interference of

tissue debris and somatic cells, a 40-micron cell filter and somatic cell lysate (0.1% SDS, 0.5% Triton X-100 in DEPC treated water) were used in sequence. The sperm was added with 10 mL of somatic cell lysate and incubated on ice for 40 minutes, after which the sperm be pelleted by centrifugation at 600 g for 15 minutes. After removal of suspension, the sperm pellet was resuspended and washed twice in 10 mL of PBS and then pelleted at 600 g for 15 minutes. Sperms were counted under a light microscope to determine the concentration.

Human sperm collection

Human sperm samples were donated by men who were visiting the Center of Reproductive Medicine, Jinling Hospital (Nanjing, China). All donors signed written informed consent forms. Sperm from aged donors (45-56 years) were enrolled as F0-Aged group, while sperm from young donors (22-27 years) were F0-Ctl group (Supplementary Table 1). Sperm samples were obtained from both group after 3–5 days of sexual abstinence. Sperm quality parameters, including concentration, motility and morphology, were evaluated according to World Health Organization guidelines (5th edition, 2010) to ensure that samples were normal.

sRNA library construction and sequencing

All sRNA library construction and sequencing were performed by BGI (Shenzhen, China). Briefly, sRNA libraries were constructed according to the TruSeq Small RNA Sample PreKit (Illumina). After library quality validation, raw data for each sRNA library were generated on the Illumina HiSeq 4000 platform. Sequence reads that fit any of the following standard quality control criterion parameters were removed: (i) reads with N (more than four bases whose quality score is lower than 10 or more than six bases whose quality score is lower than 13), (ii) reads with 5' primer contaminants or without a 3' primer, (iii) reads without the insert tag, (iv) reads with ploy A, and (v) reads shorter than 15 nt. The clean reads were obtained after data filtration.

miRNA precursor and mature sequences, tRNA and rRNA sequences were obtained from miRBase v21, GtRNAdb and National Center for Biotechnology Information, respectively. Bowtie was used to align clean reads to these reference sequences for annotation. To annotate miRNA, only candidates with one mismatch and no more than two shifts were counted as miRNA matches. To annotate tsRNA, SPORTS 1.1 based on bowtie was used for tsRNA annotation. The total sequencing frequency of each type of sRNA in each sample was normalized to 1,000,000. Differential analysis was used by Student's t test. Significance was set at uncorrected $P < 0.05$ for broad pattern identification. A fold change threshold was set at > 2. The average expression level threshold was set at > 500.

RNA isolation and quantitative RT-PCR analyses

Total RNA was isolated from sperm using TRIzol Reagent (Invitrogen, Carlsbad, CA) according to the manufacturer's instructions. To detect miRNAs, total RNA was reverse transcribed to cDNA using miRNA 1st Strand cDNA synthesis kit (Vazyme, China) and stem-loop RT primer, and sequentially incubated at 25°C for 5 minutes, 50°C for 15 minutes and 85°C for 5 minutes. Next, real-time PCR was performed using a miRNA Universal SYBR qPCR Master Mix kit (Vazyme, China) on a Light Cycler 480 real time PCR System (Roche, Mannheim, Germany). Relative miRNA expression in sperm was normalized to U6.

To detect mRNAs, total RNA was reverse transcribed to cDNA using HiScript® III RT SuperMix for qPCR (+gDNA wiper) (Vazyme, China) and corresponding RT primer, and sequentially incubated at 37°C for 15 minutes, 85°C for 5 second. Next, real-time PCR was performed using a ChamQ Universal BYBR Qpcr Master Mix kit (Vazyme, China) on a Light Cycler 480 real time PCR System (Roche, Mannheim, Germany). Relative mRNA expression in embryos and N2A cells was normalized to GAPDH.

Microinjection of sperm sRNA and miRNA mimics and transfer of embryos

Sperm sRNA was isolated by using mirVana™ miRNA Isolation kit (by Thermo Fisher Scientific). Synthetic miR-9-5p mimic and scrRNA were obtained from Ribobio (Guangzhou, China). Sequences of synthetic miRNA mimics were shown as follow: miR-9-5p mimic, 5'-UCUUUGGUUAUCUAGCUGUAUGA-3'; scrRNA, provided by Ribobio (Guangzhou, China).

sRNA or miRNA mimic was adjusted to a concentration of 2 ng/µL, which is approximately equal to the total RNA of 10 sperm. sRNA or miRNA mimic was microinjected into the cytoplasm of normal zygotes by a Leica microinjection system. After injection, the zygotes were cultured in M16 (Sigma-Aldrich) at 37°C in 5% CO_2 until to the two-cell stage embryos. Then the embryos were transferred to the oviduct of the surrogate mother (ICR background). The offspring of each group (sRNA-Aged *vs.* sRNA-Ctl; miR-9-5p *vs.* scrRNA) were screened for behavioral performances until adulthood (8-10 weeks). Approximately 300 embryos were injected for each condition, and 80 to 85% implanted embryos survived up to E3.5, 30 to 35% to E5.5, and 30 to 35% until adulthood. No difference was shown in embryo survival rate between the sRNA-Aged group and sRNA-Ctl group in each stage.

Early embryo collection and single-cell transcriptome RNA sequencing

Early embryo collection

sRNAs isolated from the sperm of F0-Aged and F0-Ctl mice or aged and young human donors were microinjected into zygotes of the C57Bl/6J background at a concentration of 2 ng/µL. The zygotes were then cultured in M16 medium (Sigma Aldrich) at 37°C in 5% CO_2. On the second day, the 2-cell embryos were transferred to potassium-supplemented simplex optimized medium (KSOM, Millipore). Embryos at the 2-cell, 4-cell, 8-cell and blastocyst stage were collected at approximately 18, 42, 76 and 96 hours after microinjection, respectively. To remove the zonapellucida, the selected embryos were transferred into an acidic solution drop (1 mL of PBS supplemented with 1 mL of 36% HCl), and all the embryos without zonapellucida were washed carefully in Dulbecco's Phosphate Buffered Saline several times to remove all the potential maternal contaminants. In addition, some 2-cell embryos were transferred to the oviduct of the surrogate mother of the ICR background. In addition, 2-cell, 4-cell, 8-cell and blastocyst embryos were pooled at each developmental stage, and total RNA was isolated from these early embryos using the Arcturus PicoPure RNA Isolation Kit (Applied Biosystems, Carlsbad, CA) according to the manufacturer's instructions for quantitative RT-PCR analysis.

Single-cell transcriptome RNA library construction

Single 8-cell-stage embryos (E2.0) and blastocyst-stage embryos (E3.5) were collected and lysed to

release all RNAs. Single-cell transcriptome RNA sequencing was performed by Anoroad (Beijing, China). After base composition and quality tests were passed, the sequence of adapters, high content of unknown bases (unknown bases more than 10%), and low-quality reads were removed. The clean reads were used for bioinformatics analysis.

miRNA target prediction and cell transfection assay

The target genes of miRNAs were predicted by TargetScan and RNAhybrid. The detailed binding sites between miRNAs and target gene was visualized as a diagram. N2A cells were cultured in Dulbecco's modified Eagle's media (DMEM, Gibco) with 10% fetal bovine serum (Gibco). For cell transfection assay, N2A cells were seeded onto 6-well plates and allowed to grow to 70% confluence, and each well was transfected with 100 pmol of miRNA mimics or scrRNA using Lipofectamine 2000 (Invitrogen) according to the manufacturer's instructions. Total RNA and protein were isolated 24 or 48 hours after transfection.

Western blot analysis

Protein samples from cells were extracted by RIPA buffer. After centrifuging at 12,000 g for 10 minutes at 4°C, the supernatant was kept for western blot analysis. BCA method was used to quantify protein concentration. The following antibodies were used: ERα (1:200; SANTA CRUZ BIOTECHNOLOGY, sc-8005), anti-G-protein coupled receptor 30 (1:250; abcam, ab39742), β-actin-HRP (1:1000; Cell Signaling Technology, 12620S). The data were quantified using ImageJ software (NIH, Bethesda, MD), and the relative protein expression was normalized to the level of β-actin.

Immunofluorescence

Embryos were collected and fixed in 4% paraformaldehyde for 30 minutes at room temperature. After being washed with PBST (containing 0.1% Triton X-100 and 0.1% Tween 20), they were incubated by PBS including 1% BSA and 0.1% Tween 20 for 60 minutes. Embryos were incubated overnight at 4°C with the following primary antibodies: Anti-G-protein coupled receptor 30 (1:200, abcam), β-Actin (D6A8) Rabbit mAb (HRP Conjugate). After a complete wash in PBS, the embryos were incubated in the appropriate secondary antibodies for 2 hours at room temperature in the dark. The embryos were washed and mounted in Fluoromount-G mounting medium (SouthernBiotech, Birmingham, AL). The micrographs were taken by inverted laser scanning confocal microscope (TCS SP8, Leica). Digital images were recorded and analyzed by LAS X Viewer software (Leica).

Statistical analysis

Statistical analyses were performed using the Prism v.8.0 (GraphPad Software Inc.). The D'Agostino & Pearson normality test was used to measure data distribution. Two-tailed unpaired Student's t test was conducted to compare two groups. Data are presented as the means ± standard errors of the means (SEMs). Differences are considered statistically significant at $P < 0.05$. *$P < 0.05$; **$P < 0.01$; ***$P < 0.001$.

Supplementary Figures

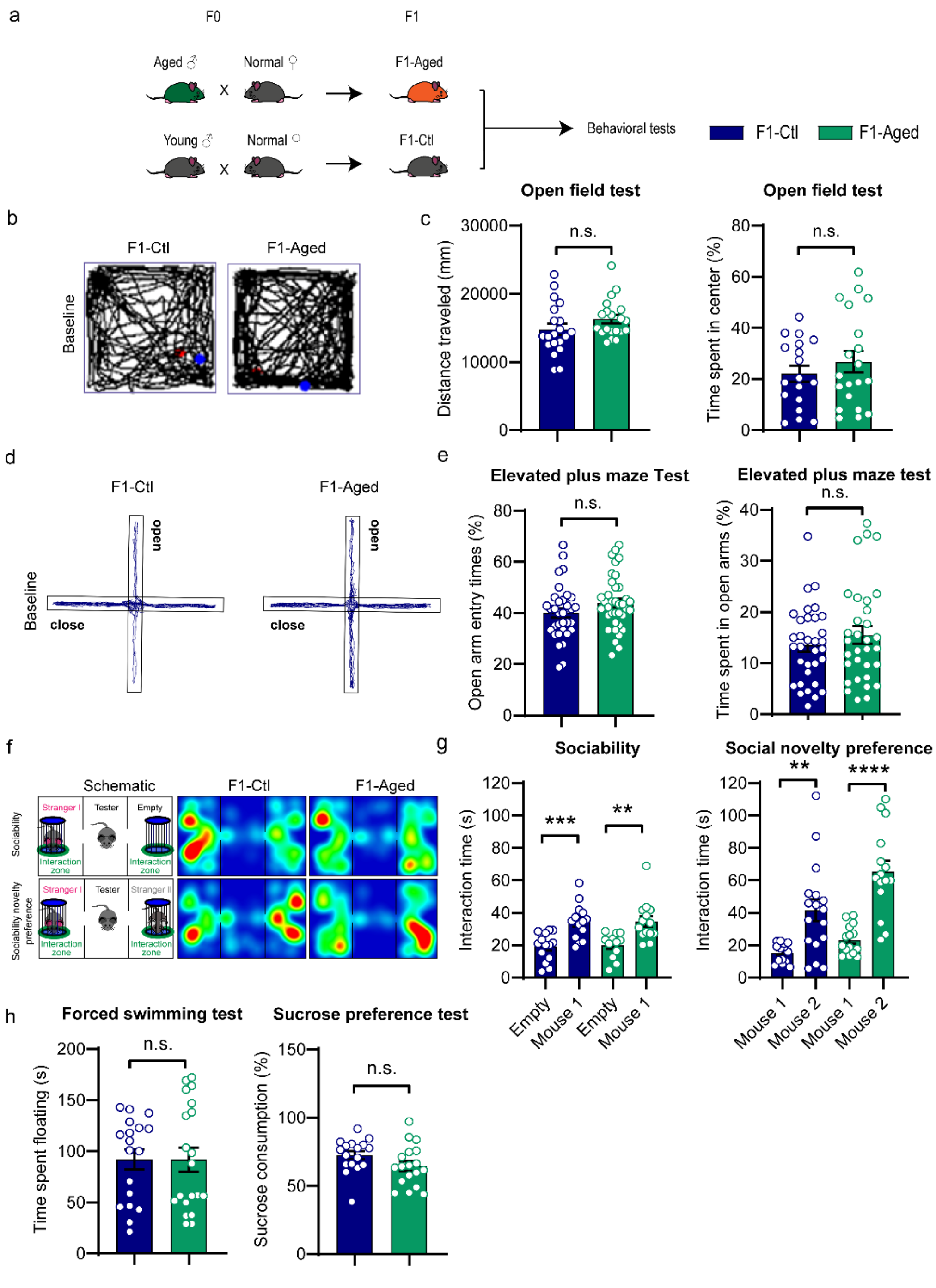

Supplementary Fig. S1. The offspring of aged male mice show no behavioral abnormalities in basal conditions. (a) Breeding scheme of aged father (F0-Aged *vs.* F0-Ctl) and behavioral paradigm in F1 offspring (F1-Aged *vs.* F1-Ctl) under baseline condition. **(b-c)** Open-field test. Representative sample tracks of mouse movement in the open-field test, total distance traversed in the open arena, and percent time spent in central areas of the arena (n = 18-20 per group). **(d-e)** Elevated plus maze test. Representative sample tracks of mouse movement in the elevated plus maze test, percent number of entries into open arms, and percent time spent in the open arms (n = 32-37 per group).

(f-g) Three-chamber social interaction test. Heat map showing the amount of time spent in the three-chamber arena at the sociability and social novelty preference portion of the tests, total time spent sniffing the stranger mouse 1 and empty, and total time spent sniffing the stranger mouse 1 and mouse 2 (n = 12-17 per group). **(h)** Behavioral performances in forced swimming test (n = 18-20 per group) and sucrose preference test (n = 17-18 per group).

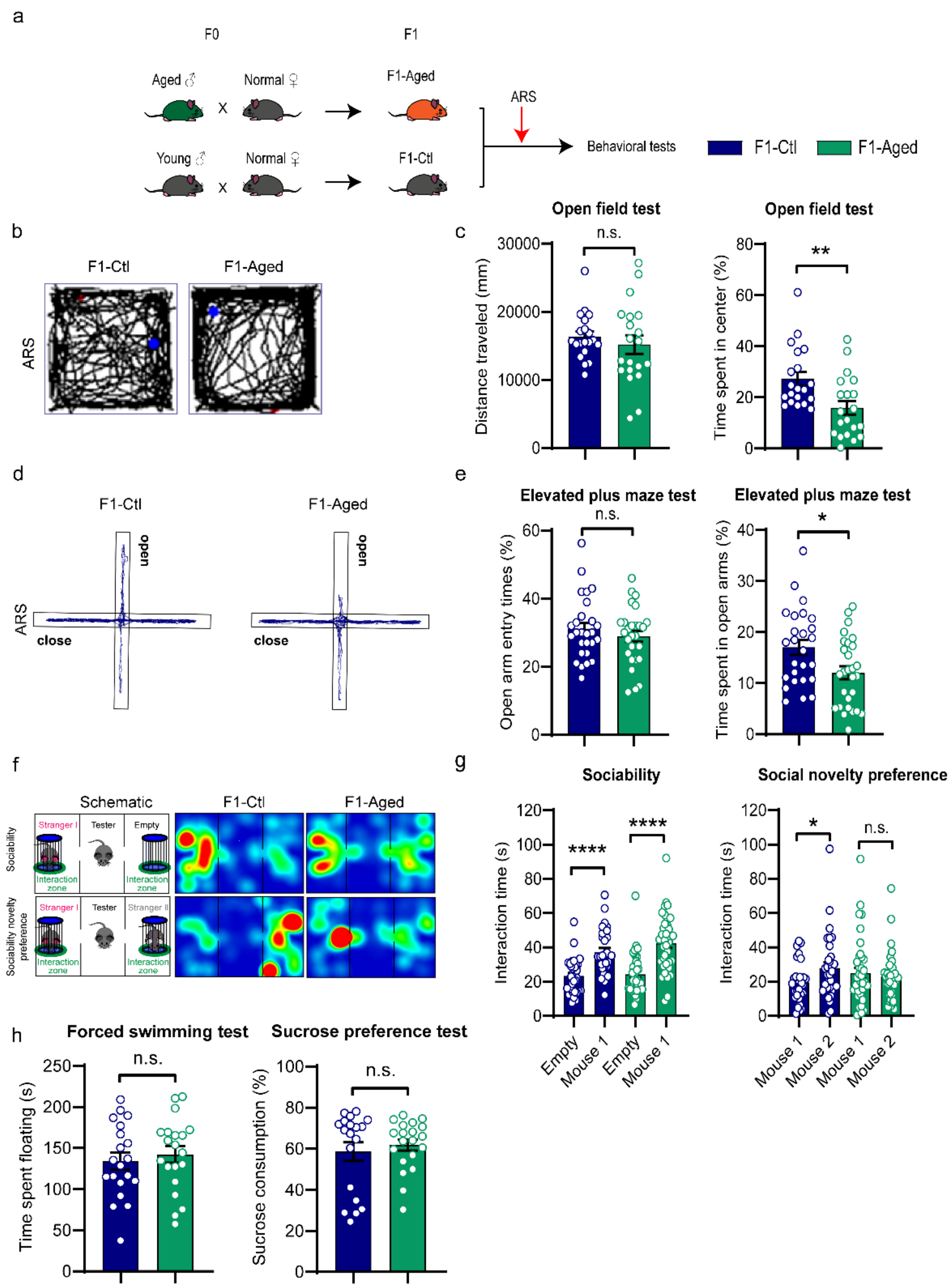

Supplementary Fig. S2. Aged paternal pregnancy leads to susceptibility of anxiety-like and social disorder in offspring. (a) Breeding scheme of aged father (F0-Aged *vs.* F0-Ctl) and behavioral paradigm in F1 offspring (F1-Aged *vs.* F1-Ctl) after exposure to ARS. **(b-c)** Open-field test. Representative sample tracks of mouse movement in the open-field test, total distance traversed in the open arena, and percent time spent in central areas of the arena (n = 20 per group). **(d-e)** Elevated plus maze test. Representative sample tracks of mouse movement in the elevated plus maze test, percent number of entries into open arms, and percent time spent in the open arms (n = 26-27 per group). **(f-g)** Three-chamber social interaction test. Heat map showing the amount of time spent

in the three-chamber arena at the sociability and social novelty preference portion of the tests, total time spent sniffing the stranger mouse 1 and empty, and total time spent sniffing the stranger mouse 1 and mouse 2 (n = 31 per group). **(h)** Behavioral performances in forced swimming test (n = 20 per group) and sucrose preference test (n = 19-20 per group).

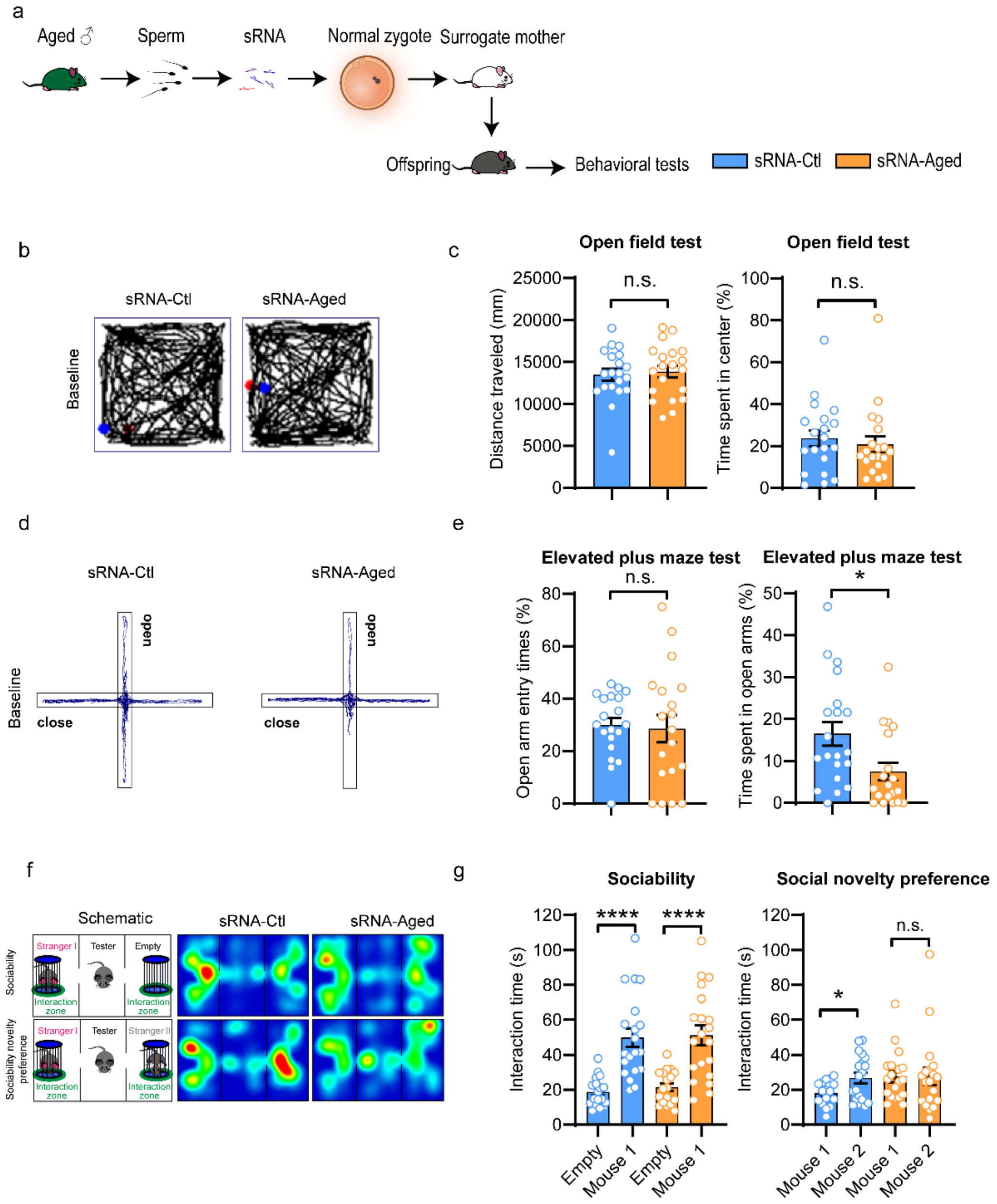

Supplementary Fig. S3. The IVF offspring born to zygotes injected with sperm sRNA derived from aged male mice show no behavioral abnormalities in basal conditions. (a) Schematic timeline and behavioral paradigm in IVF offspring born to zygotes injected with sperm sRNA (sRNA-Aged *vs.* sRNA-Ctl) under baseline condition. **(b-c)** Open-field test. Representative sample tracks of mouse movement in the open-field test, total distance traversed in the open arena, and percent time spent in central areas of the arena (n = 20 per group). **(d-e)** Elevated plus maze test. Representative sample tracks of mouse movement in the elevated plus maze test, percent number of entries into open arms, and percent time spent in the open arms (n = 19-20 per group). **(f-g)** Three-chamber social interaction test. Heat map showing the amount of time spent in the three-chamber arena at the sociability and social novelty preference portion of the tests, total time spent sniffing

the stranger mouse 1 and empty, and total time spent sniffing the stranger mouse 1 and mouse 2 (n = 16-20 per group).

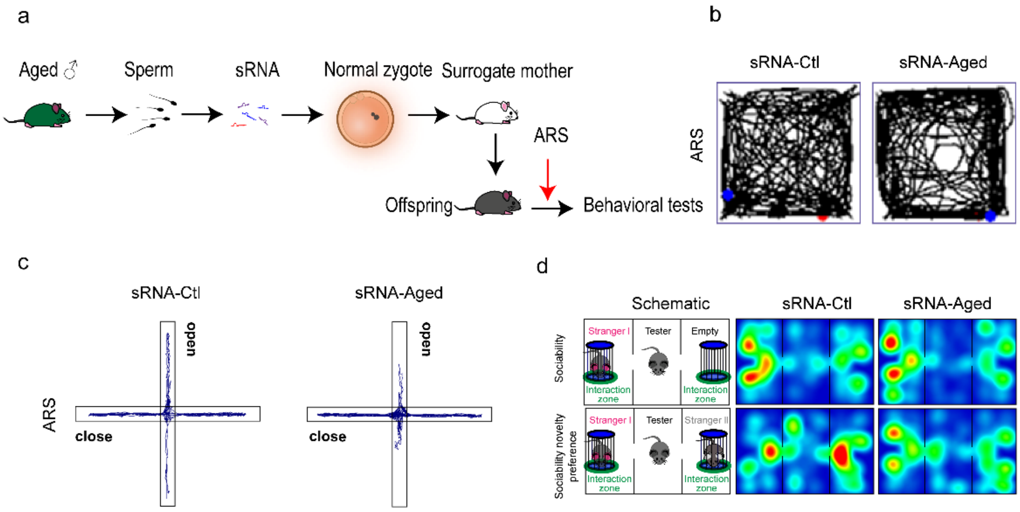

Supplementary Fig. S4. Sperm sRNAs confer aging-induced susceptibility of anxiety-like and social disorder to offspring. (a) Schematic timeline and behavioral paradigm in IVF offspring born to zygotes injected with sperm sRNA (sRNA-Aged *vs.* sRNA-Ctl) after exposure to ARS. **(b)** Open-field test. Representative sample tracks of mouse movement in the open-field test. **(c)** Elevated plus maze test. Representative sample tracks of mouse movement in the elevated plus maze test. **(d)** Three-chamber social interaction test. Heat map showing the amount of time spent in the three-chamber arena at the sociability and social novelty preference portion of the tests.

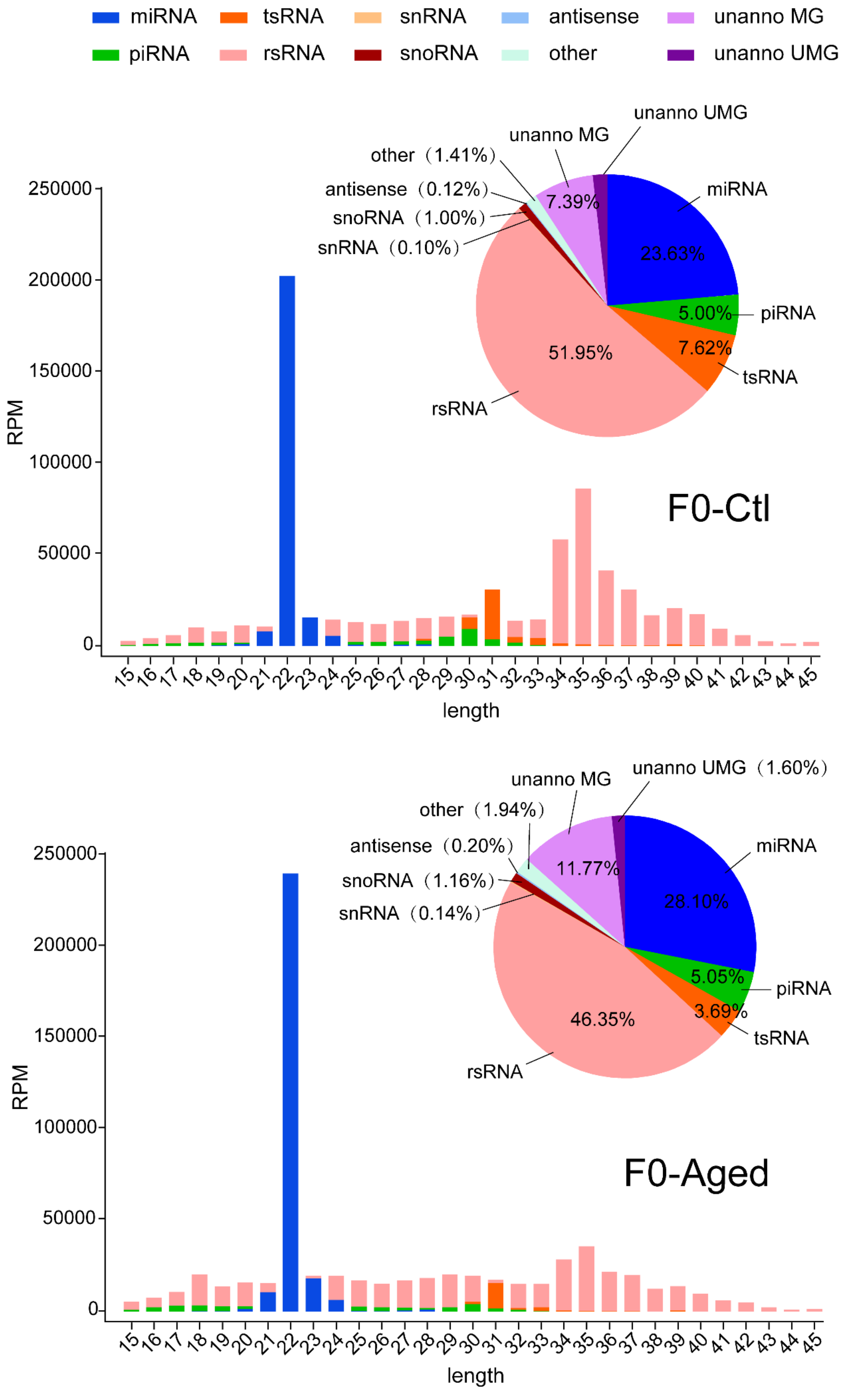
miRNA
tsRNA
snRNA
antisense
unanno MG
piRNA
rsRNA
snoRNA
other
unanno UMG
F0-Ctl
RPM
length
unanno UMG
unanno MG
other（1.41%）
antisense（0.12%）
snoRNA（1.00%）
snRNA（0.10%）
7.39%
miRNA
23.63%
5.00%
piRNA
7.62%
tsRNA
51.95%
rsRNA
F0-Aged
unanno UMG（1.60%）
unanno MG
other（1.94%）
antisense（0.20%）
snoRNA（1.16%）
snRNA（0.14%）
11.77%
28.10%
5.05%
3.69%
46.35%

Supplementary Fig. S5. Annotation and classification of the sRNA species in the sperm derived from F0-Aged and F0-Ctl. Sperm sRNA fragments of 10-50 bp were isolated from the gel and processed for sRNA deep sequencing. The sequence reads were annotated and classified through alignment with known sRNA libraries. The y-axis shows the reads relative to per million total reads. Classification of the sRNA species revealed that rsRNA, miRNA, piRNA and tsRNA were the most abundant sRNAs in sperm, which accounted for 46.35%, 28.10%, 5.05% and 3.69% in the sperm of F0-Aged and 51.95%, 23.63%, 5.00% and 7.62% in the sperm of F0-Ctl, respectively. Length distribution analysis showed a dominant distribution of miRNAs at 22 bp and tsRNA at 31 bp and a widespread distribution of rsRNA at 15-45 bp and piRNA at 15-32 bp. Unanno MG: matched to the reference genome but not annotated. Unanno UMG: unmatched to the reference genome and not annotated.

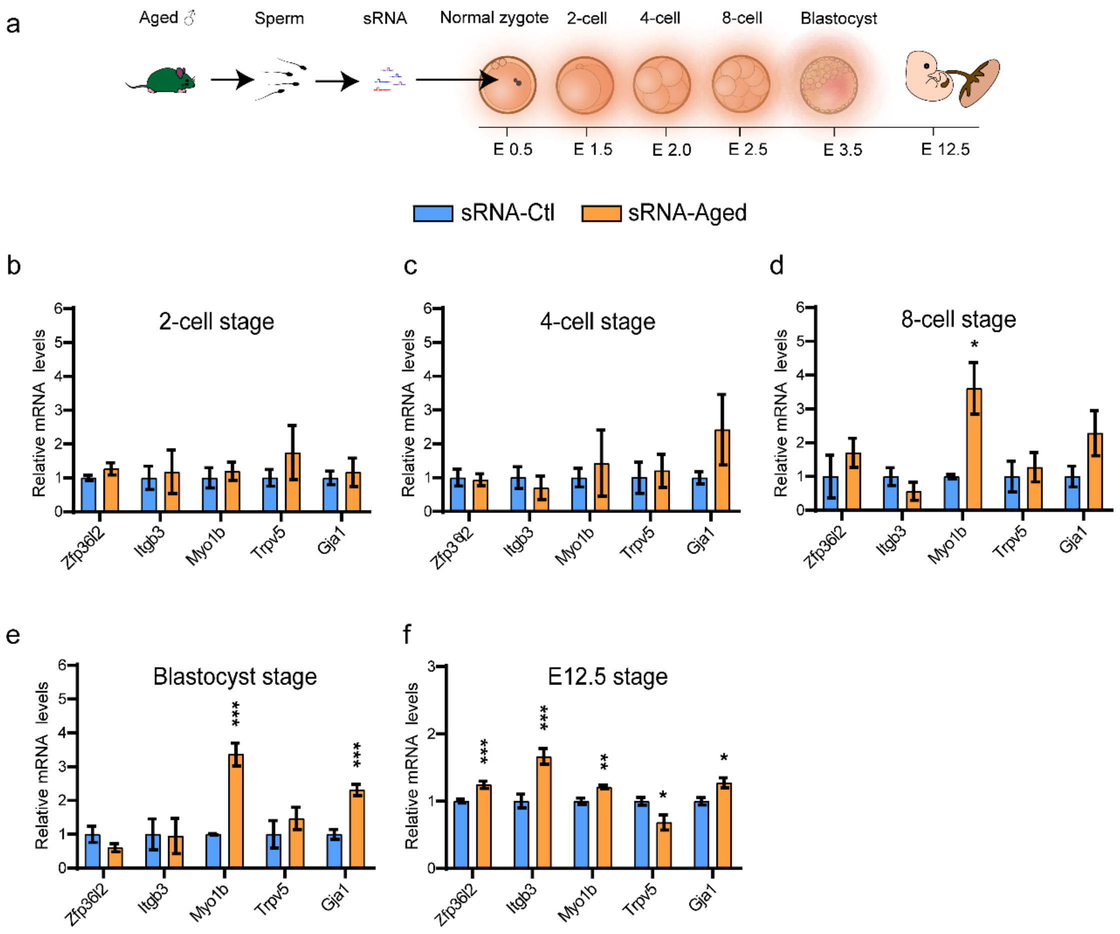

Supplementary Fig. S6. Alteration of the genes (Zfp36l2, Itgb3, Myo1b, Trpv5 and Gja1) downstream the 17β-estradiol signaling during early embryonic development. (a) Schematic timeline in IVF offspring born to zygotes injected with sperm sRNA (sRNA-Aged *vs.* sRNA-Ctl). **(b-f)** Quantitative RT-PCR analysis of the expression levels of Zfp36l2, Itgb3, Myo1b, Trpv5 and Gja1 mRNAs at 2-cell (b), 4-cell (c), 8-cell (d), blastocyst (e) and E12.5 (f) stages (n = 3 per group).

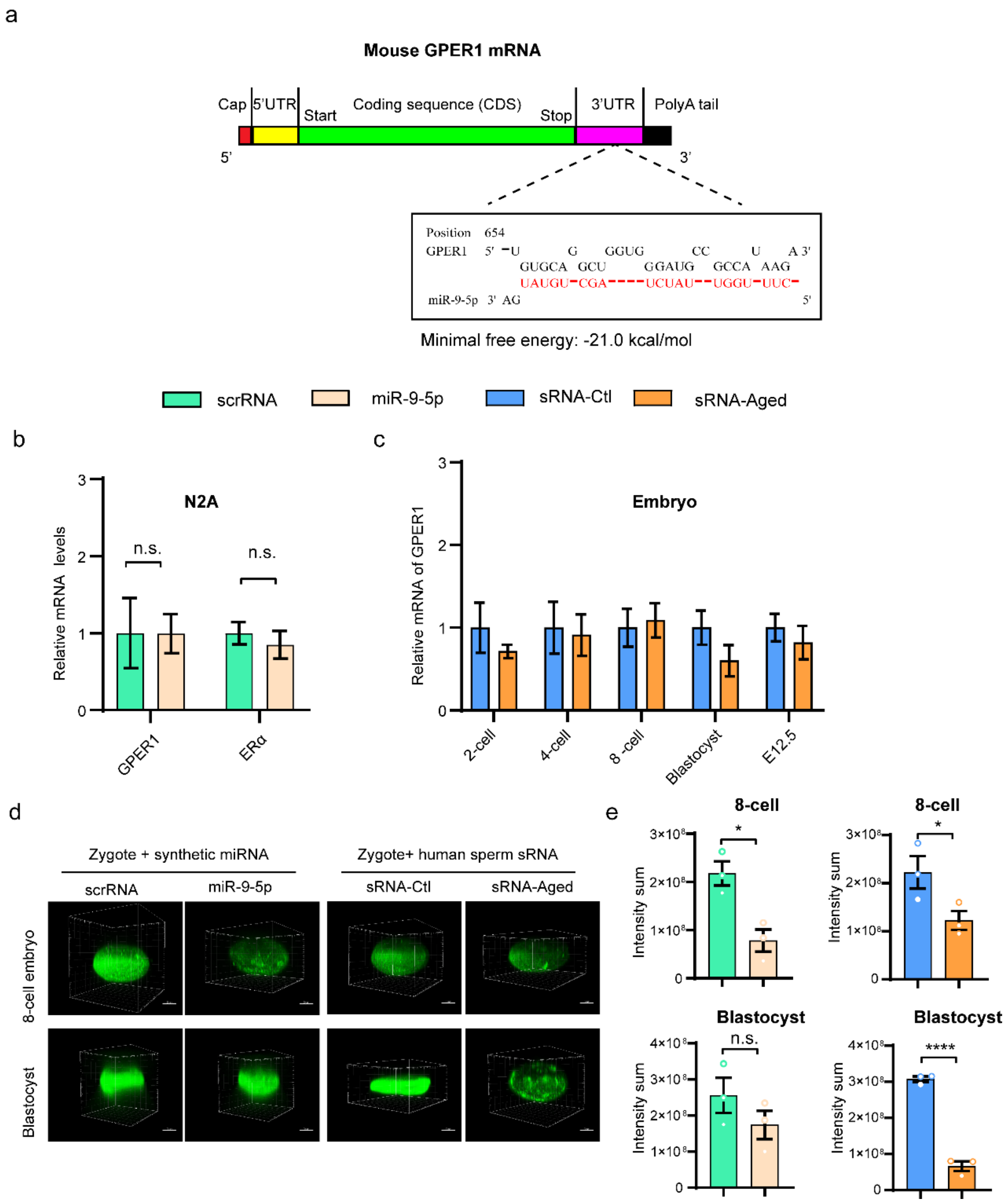

Supplementary Fig. S7. miR-9-5p from aged sperm causes GPER1 downregulation during early embryonic development. (a) Diagram of the putative binding of miR-9-5p on the 3'-UTR of GPER1. **(b)** Quantitative RT-PCR analysis of the expression levels of GPER1 and ERα mRNAs in N2A cells transfected with scrambled RNA (scrRNA) or miR-9-5p (n = 6 per group). **(c)** Sperm sRNAs (sRNA-Aged *vs.* sRNA-Ctl) were injected into zygotes, and the alteration of GPER1 mRNA was assessed by quantitative RT-PCR when the embryos developed to 2-cell, 4-cell, 8-cell, blastocyst and E12.5 stages (n = 5 per group). **(d)** Immunofluorescence analysis of GPER1 in the 8-cell embryos and blastocysts developing from zygotes injected with miR-9-5p or scrRNA (left), or in the 8-cell embryos and blastocysts developing from zygotes injected with sperm sRNAs from aged or young human donors (right). **(e)** Quantification of the immunofluorescence staining intensity (n = 3 per group).

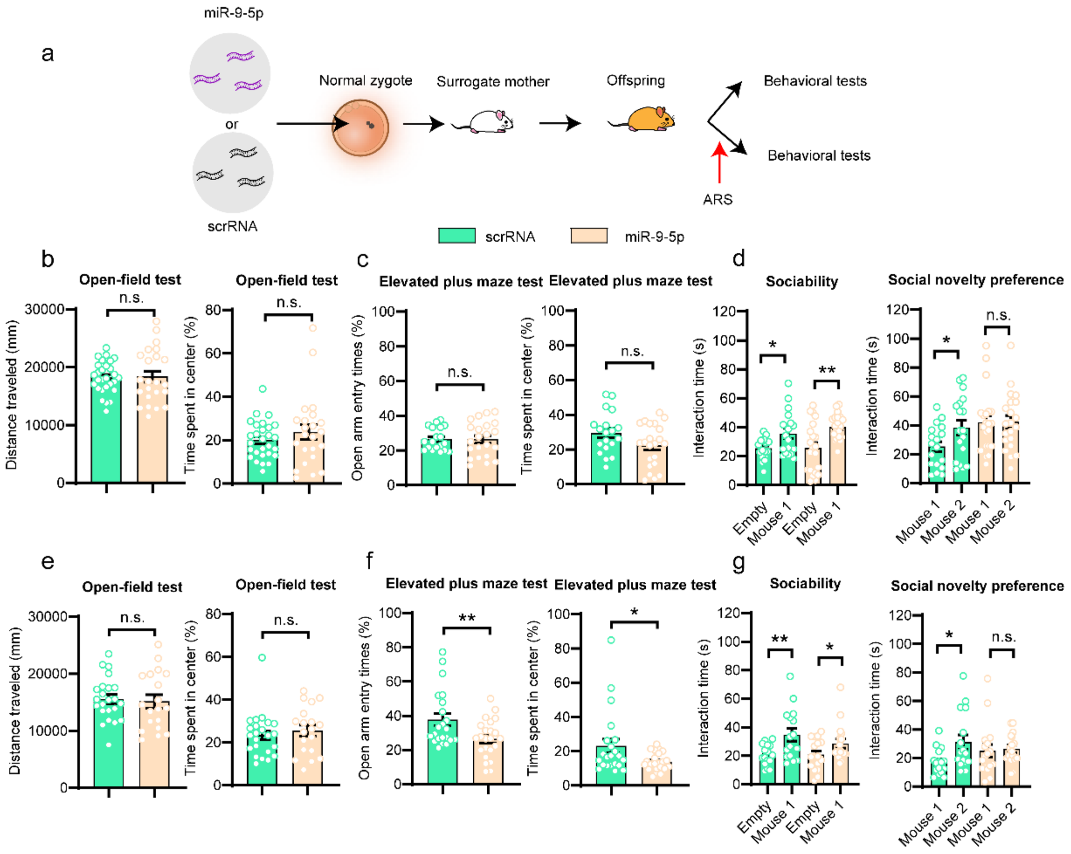

Supplementary Fig. S8. The IVF offspring born to zygotes injected with miR-9-5p show anxiety-like behaviors and social disorder. (a) Schematic timeline and behavioral paradigm in IVF offspring born to zygotes injected with synthetic miRNAs (miR-9-5p *vs.* scrRNA) under baseline condition or after exposure to ARS. **(b)** Open-field test in basal conditions: total distance traversed in the open arena (left), and percent time spent in central areas of the arena (right) (n = 23-28 per group). **(c)** Elevated plus maze test in basal conditions: percent number of entries into open arms (left), and percent time spent in the open arms (right) (n = 19-22 per group). **(d)** Three-chamber social interaction test in basal conditions: total time spent sniffing the stranger mouse 1 and empty (left), and total time spent sniffing the stranger mouse 1 and mouse 2 (right) (n = 18-21 per group). **(e)** Open-field test after exposure to ARS: total distance traversed in the open arena (left), and percent time spent in central areas of the arena (right) (n = 19-23 per group). **(f)** Elevated plus maze test after exposure to ARS: percent number of entries into open arms (left), and percent time spent in the open arms (right) (n = 22-23 per group). **(g)** Three-chamber social interaction test after exposure to ARS: total time spent sniffing the stranger mouse 1 and empty (left), and total time spent sniffing the stranger mouse 1 and mouse 2 (right) (n = 16-17 per group).

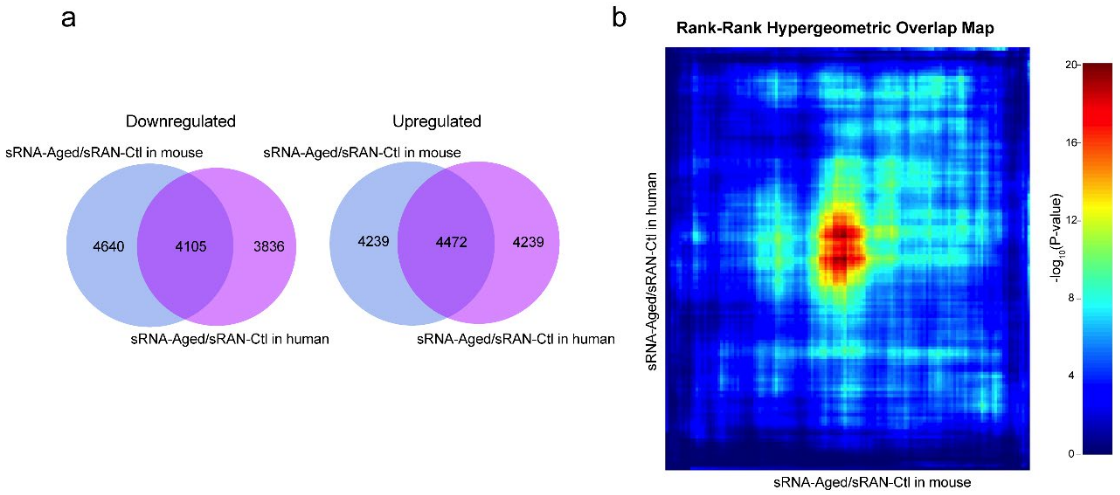

Supplementary Fig. S9. Comparison of the transcriptional changes in blastocysts induced by human and mouse sperm sRNAs. Sperm sRNAs derived from aged and young human donors were injected into zygotes, and the alteration of gene profiles was assessed by single-cell transcriptome RNA sequencing when the embryos developed to blastocyst stage. Then the transcriptional changes induced by human sperm sRNAs were compared with those induced by mouse sperm sRNAs (sRNA-Aged/sRNA-Ctl in human *vs.* sRNA-Aged/sRNA-Ctl in mouse). **(a)** A Venn diagram depicting a large overlap of the transcripts significantly upregulated or downregulated in the blastocysts developed from the zygotes injected with human and mouse sperm sRNAs. **(b)** Comparison of the differentially expressed genes by rank-rank hypergeometric overlap (RRHO) analysis (which estimates the similarities between two ranked lists). RRHO analysis shows a coregulated pattern in the blastocysts developed from the zygotes injected with human and mouse sperm sRNAs. The pixels represent the overlap between the transcriptome of each comparison; the significance of the overlap [$-\log_{10}$ (P value) of a hypergeometric test] is color coded. Genes along each axis are sorted from most significantly upregulated to most downregulated from the lower left corner.

Supplementary Table 1. Donor information for sperm samples.

No.	Gender	Age (years)	Group
1	male	27	Young
2	male	25	Young
3	male	26	Young
4	male	23	Young
5	male	25	Young
6	male	27	Young
7	male	23	Young
8	male	22	Young
9	male	26	Young
10	male	23	Young
11	male	22	Young
12	male	23	Young
13	male	24	Young
14	male	26	Young
15	male	24	Young
16	male	26	Young
17	male	22	Young
18	male	22	Young
19	male	25	Young
20	male	27	Young
21	male	26	Young
22	male	23	Young
23	male	27	Young
24	male	46	Aged
25	male	45	Aged
26	male	52	Aged
27	male	50	Aged
28	male	56	Aged
29	male	45	Aged
30	male	45	Aged
31	male	54	Aged
32	male	47	Aged
33	male	45	Aged
34	male	47	Aged
35	male	56	Aged
36	male	50	Aged
37	male	48	Aged
38	male	46	Aged

39	male	47	Aged
40	male	45	Aged
41	male	50	Aged
42	male	49	Aged
43	male	51	Aged
44	male	46	Aged
45	male	45	Aged
46	male	53	Aged

Supplementary Table 2. Information for the top 100 DEGs between sRNA-Aged and sRNA-Ctl at 8-cell stage.

8-cell stage					
Gene ID	Name	Mean level		log_2FoldChange	p-value
		sRNA-Ctl	sRNA-Aged		
ENSMUSG00000074166	AW146154	43.5	0	8.03527599	0.00031
ENSMUSG00000001525	Tubb5	773.25	154	2.540858475	0.000441
ENSMUSG00000028807	Zbtb8a	639.25	268.25	1.455611051	0.000312
ENSMUSG00000097695	Gm26905	29	2.75	3.584040784	0.000512
ENSMUSG00000027424	Mgme1	1327.75	766	0.960798602	6.01E-05
ENSMUSG00000068206	Pick1	186.5	92.75	1.161745929	0.000557
ENSMUSG00000101257	2310015K22Rik	297.5	37	3.121175539	6.25E-05
ENSMUSG00000062510	Nsl1	765.25	427	0.996419636	0.000399
ENSMUSG00000020910	Adprm	32	0	7.574582911	0.000492
ENSMUSG00000099021	Rn7s1	39.5	0	7.888747088	9.95E-05
ENSMUSG00000049670	Morn4	37.5	0	7.814997771	0.000128
ENSMUSG00000062190	Lancl2	34	0	7.668101202	0.000304
ENSMUSG00000042628	Zfyve1	710.75	217.25	1.872862322	5.26E-06
ENSMUSG00000046133	C130073F10Rik	3459.25	1182.5	1.69784479	1.42E-05
ENSMUSG00000034220	Gpc1	20.25	0	6.982996316	0.000357
ENSMUSG00000020766	Galk1	1307.75	721.75	1.054519694	0.000513
ENSMUSG00000004896	Rrnad1	395.75	153.25	1.562856711	9.58E-07
ENSMUSG00000062070	Pgk1	506.5	313.5	0.881069036	0.000249
ENSMUSG00000097483	Gm26806	47.25	0.25	7.464423132	1.01E-08
ENSMUSG00000102630	Gm37289	21.5	0.25	6.32945587	1.92E-05
ENSMUSG00000072295	C2cd6	16.25	0	6.641913076	8.15E-05
ENSMUSG00000086359	9630013K17Rik	14.5	0	6.475829183	0.000229
ENSMUSG00000013629	Cad	964.25	676.25	0.699228315	6.69E-05
ENSMUSG00000037731	Themis2	38.5	0	7.896635383	1.58E-08
ENSMUSG00000021918	Nek4	14	0	6.436720454	0.000357

ENSMUSG00000029920	Smarcad1	3095.5	2163.75	0.689117642	0.000301
ENSMUSG00000033712	Ccar2	190.75	59.75	1.812728774	0.000343
ENSMUSG00000008373	Prpf31	2812.25	2067.75	0.625276343	0.000264
ENSMUSG00000026669	Mcm10	4669.5	2352.25	1.161118139	0.000316
ENSMUSG00000031832	Taf1c	927.5	488.75	1.082262719	0.000138
ENSMUSG00000021550	2210016F16Rik	2337.75	1295	1.028584051	0.000459
ENSMUSG00000029439	Sfswap	394	149.75	1.531340213	0.000415
ENSMUSG00000030443	Zfp583	196.5	0	10.20893284	2.55E-15
ENSMUSG00000039813	Tbc1d2	68.5	0	8.701397822	4.65E-12
ENSMUSG00000086605		38.75	0.25	7.148177495	3.40E-07
ENSMUSG00000083161	Gm11427	57.5	0.5	7.040137975	1.34E-07
ENSMUSG00000042632	Pla2g6	78.75	16	2.53167898	0.000416
ENSMUSG00000020689	Itgb3	0	39.25	-7.462327993	9.56E-05
ENSMUSG00000037492	Zmat4	0	35	-7.300344094	0.000106
ENSMUSG00000028699	Tspan1	282.25	1054.25	-1.664409943	0.000182
ENSMUSG00000049939	Lrrc4	0	27.25	-6.995412612	0.000551
ENSMUSG00000045034	Ankrd34b	0.25	56.25	-7.287955527	0.00057
ENSMUSG00000050777	Tmem37	548.5	1760.25	-1.488015142	1.49E-05
ENSMUSG00000042035	Igsf3	85.5	412	-2.065477824	0.000505
ENSMUSG00000035958	Tdp2	1732	3360.5	-0.779383941	0.000145
ENSMUSG00000025962	Fastkd2	1129.75	2261.25	-0.808749972	2.23E-05
ENSMUSG00000093930	Hmgcs1	830.5	1699.5	-0.846871813	0.000206
ENSMUSG00000021411	Pxdc1	0	36.25	-7.403560727	3.01E-05
ENSMUSG00000042073	Abhd14b	0	40	-7.530939885	0.000282
ENSMUSG00000035522	Tsga8	53.5	1043	-3.995428666	2.69E-08
ENSMUSG00000000938	Hoxa10	0	46.5	-7.730735719	0.000172
ENSMUSG00000023906	Cldn6	226	1070	-2.068392071	0.000143
ENSMUSG00000035798	Zdhhc17	19.25	152.75	-2.792360244	3.38E-06
ENSMUSG00000096992	Gm26788	0	24	-6.804523439	5.09E-05
ENSMUSG00000028098	Rnf115	1576.25	2972.25	-0.727110036	5.28E-05
ENSMUSG00000034413	Neurl1b	0	37.5	-7.48560575	0.000328
ENSMUSG00000030465	Psd3	0	34.5	-7.409984227	0.000496
ENSMUSG00000059654	Reg1	35.25	164.5	-2.081331091	8.04E-05
ENSMUSG00000074183	Gsta1	296	947.25	-1.534974656	0.000187
ENSMUSG00000022125	Cln5	169.5	499.25	-1.415953088	0.000294
ENSMUSG00000022176	Rem2	0	42.5	-7.729841099	0.000293
ENSMUSG00000052949	Rnf157	0	34.5	-7.405462559	0.000453
ENSMUSG00000005893	Nr2c2	620.25	1619	-1.220911758	0.000544
ENSMUSG00000030499	Kctd15	16	224.25	-3.632549833	6.44E-05
ENSMUSG00000031530	Dusp4	66	373.25	-2.320588453	3.98E-06
ENSMUSG00000045817	Zfp36l2	255.25	697.25	-1.275666552	0.000187
ENSMUSG00000053025	Sv2b	0	47.5	-7.871256201	0.000151
ENSMUSG00000027351	Spred1	268	1062	-1.858813204	2.05E-05
ENSMUSG00000048562	Sp8	0	75.25	-8.615713327	3.10E-05

ENSMUSG00000061455	Stx17	1329.75	2687.25	-0.840313537	0.000131
ENSMUSG00000019866	Crybg1	143.25	640.75	-1.991398397	0.000503
ENSMUSG00000028382	Ptbp3	756.75	1809.5	-1.067823014	3.33E-05
ENSMUSG00000022797	Tfrc	14021	25348.5	-0.665481601	0.000493
ENSMUSG00000024378	Stard4	789.75	1841	-1.062045731	7.26E-05
ENSMUSG00000054252	Fgfr3	56.25	371.25	-2.579487839	3.81E-06
ENSMUSG00000015619	Gata3	64.25	834.75	-3.537435716	1.27E-07
ENSMUSG00000103039	Gm37123	0.5	24.25	-5.413412389	0.000485
ENSMUSG00000022766	Serpind1	0	18.5	-6.456774161	0.000147
ENSMUSG00000025439	Clns1a	14338.5	23927.25	-0.565357784	1.33E-06
ENSMUSG00000091931	Gon7	604	1064.5	-0.645018557	2.40E-05
ENSMUSG00000030207	Fam234b	52.5	220	-1.871895009	1.07E-05
ENSMUSG00000009900	Wnt3a	0.75	40.25	-5.522629661	2.41E-05
ENSMUSG00000027035	Cers6	955.75	3764.75	-1.784838848	1.77E-07
ENSMUSG00000019818	Cd164	5343.5	8772	-0.53256024	0.000289
ENSMUSG00000028518	Prkaa2	118.5	471.5	-1.800075199	6.09E-06
ENSMUSG00000006527	Sfmbt1	94.25	305.25	-1.501777653	8.73E-05
ENSMUSG00000030990	Pgap2	1006.75	1822.25	-0.688489048	0.000462
ENSMUSG00000042599	Kdm7a	614.75	1535.75	-1.152611379	7.47E-07
ENSMUSG00000020608	Smc6	2215.25	3469.75	-0.472536629	0.000375
ENSMUSG00000098015	Gm44527	0	63.25	-8.306228325	3.02E-09
ENSMUSG00000098650	Gm28048	0	51.25	-7.999072448	8.14E-08
ENSMUSG00000020515	Cnot8	6193	10149.75	-0.537373604	0.00027
ENSMUSG00000069910	Spdl1	1338.5	2484	-0.718280537	0.000432
ENSMUSG00000057706	Mex3b	100.75	481.5	-2.128213896	1.30E-06
ENSMUSG00000033629	Hacd3	625	1700.25	-1.293120193	7.31E-05
ENSMUSG00000016239	Lonrf3	257.25	822.75	-1.513949784	0.000227
ENSMUSG00000030660	Pik3c2a	1191	2896.75	-1.123981467	5.80E-05
ENSMUSG00000049606	Zfp644	772.75	2444.5	-1.495225082	7.39E-05
ENSMUSG00000062421	Arf2	867.75	1808.75	-0.900615403	0.000293
ENSMUSG00000020986	Sec23a	1655.25	3043	-0.707260255	0.000423

Supplementary Table 3. Information for the top 100 DEGs between sRNA-Aged and sRNA-Ctl at blastocyst stage.

Blastocyst stage					
**Gene ID**	**Name**	**Mean level**		**log_2FoldChange**	**p-value**
		**sRNA-Ctl**	**sRNA-Aged**		
ENSMUSG00000029385	Ccng2	376.25	18.5	4.449090945	6.59E-11
ENSMUSG00000103891	Gm37941	122	3	5.422283525	2.37E-09
ENSMUSG00000032446	Eomes	1789.25	425.25	2.166752515	2.84E-11

ENSMUSG00000024727	Trpm6	1289.75	641.5	1.098993356	6.29E-09
ENSMUSG00000051343	Rab11fip5	878	263	1.831723351	2.03E-09
ENSMUSG00000038780	Smurf1	839.75	327.25	1.453192751	4.52E-11
ENSMUSG00000075284	Wipf1	1348	506.25	1.507088762	7.6E-09
ENSMUSG00000042680	Garem1	640.5	252.75	1.437603198	2.65E-09
ENSMUSG00000024922	Ovol1	512	167.75	1.693845391	4.06E-12
ENSMUSG00000092274	Neat1	3768.75	1354.25	1.562628928	8.65E-10
ENSMUSG00000050628	Ubald2	1313.25	583.25	1.268902552	4.88E-09
ENSMUSG00000026743	Mllt10	2126.25	1050	1.115427101	1.97E-12
ENSMUSG00000019854	Reps1	2554.75	636.25	2.109731066	5.2E-15
ENSMUSG00000031410	Nxf7	1618.25	407.5	2.098127101	4.36E-12
ENSMUSG00000021361	Tmem14c	4715	2021.75	1.322804599	2.8E-09
ENSMUSG00000046432	Bex3	5928.5	1330	2.269486684	4.76E-08
ENSMUSG00000072770	Acrbp	389.75	142	1.552122594	3.99E-08
ENSMUSG00000028699	Tspan1	725.25	137.25	2.507656506	1.07E-12
ENSMUSG00000020656	Grhl1	1043.5	235.5	2.243900186	1.31E-12
ENSMUSG00000028469	Npr2	414.5	92	2.273800931	9.36E-10
ENSMUSG00000022686	B3gnt5	8341.75	2102.75	2.090899855	1.54E-10
ENSMUSG00000030660	Pik3c2a	2652.25	839	1.762217858	1.18E-08
ENSMUSG00000078816	Prkcg	2275.5	793	1.619624982	1.99E-08
ENSMUSG00000026600	Soat1	8678.5	2166.25	2.104030698	4.32E-09
ENSMUSG00000021792	Fam213a	7090.75	1915.75	1.978321662	4.95E-12
ENSMUSG00000027954	Efna1	483.75	62	3.069194955	3.97E-08
ENSMUSG00000029864	Gstk1	612.75	106.75	2.628122715	7.14E-14
ENSMUSG00000037370	Enpp1	1048.5	289	1.962747609	2.21E-09
ENSMUSG00000042524	Sun2	49	1.25	5.400704995	5.34E-09
ENSMUSG00000044308	Ubr3	1151.25	644	0.933966772	4.68E-08
ENSMUSG00000034394	Lif	840	209.75	2.101889199	6.61E-12
ENSMUSG00000028173	Wls	1571.5	201.5	3.07271818	9.78E-11
ENSMUSG00000037989	Wnk2	1567.75	507.5	1.734321642	1.63E-08
ENSMUSG00000022899	Slc15a2	8973.25	3084	1.646862339	1.76E-11
ENSMUSG00000030849	Fgfr2	3370.5	1693.5	1.092879692	1.24E-08
ENSMUSG00000078453	Abracl	3263.5	680.75	2.380305985	5.39E-08
ENSMUSG00000003161	Sri	8949	2241.75	2.108200657	1.33E-10
ENSMUSG00000028716	Pdzk1ip1	2526.75	760.75	1.844416964	2.53E-08
ENSMUSG00000063652	Slc22a21	501.25	105	2.335292944	1.9E-14
ENSMUSG00000022900	Ildr1	1904	1031	0.976854108	2.12E-09
ENSMUSG00000048490	Nrip1	464.25	163	1.604716917	7.44E-12
ENSMUSG00000069114	Zbtb10	4208.5	1922.75	1.22796857	1.26E-11
ENSMUSG00000018554	Ybx2	237.75	70	1.850707091	4.46E-11
ENSMUSG00000011256	Adam19	386	71.75	2.522800068	1.52E-18
ENSMUSG00000032575	Manf	6699	2449.5	1.545766935	7.61E-17
ENSMUSG00000047767	Atg16l2	1360.25	590.25	1.297937752	1.55E-11
ENSMUSG00000030177	Ccdc77	2549.75	1301.75	1.065094776	1.12E-08

ENSMUSG00000001999	Blvra	2616.25	1115.75	1.332810236	0.00000002
ENSMUSG00000026548	Slamf9	640.25	230.75	1.57130796	3.82E-11
ENSMUSG00000034007	Scaper	729.25	333	1.233297277	1.97E-08
ENSMUSG00000009092	Derl3	65.25	4.75	3.885889395	1.78E-09
ENSMUSG00000030792	Dkkl1	10427	3977.75	1.496720704	2.09E-09
ENSMUSG00000028602	Tnfrsf8	17	161.75	-3.164125437	1.04E-08
ENSMUSG00000044229	Nxpe4	0	41.75	-7.738983201	3.90E-09
ENSMUSG00000041548	Hspb8	73	494.5	-2.690800896	5.52E-09
ENSMUSG00000040717	Il17rd	6.5	80	-3.54530257	5.44E-08
ENSMUSG00000037335	Hand1	0	266	-10.43620714	6.38E-12
ENSMUSG00000026510	Trp53bp2	191	725	-1.839540775	5.12E-09
ENSMUSG00000020114	Cand1	1962.5	5167.5	-1.304308401	0.000000013
ENSMUSG00000032030	Cul5	1845.25	6696	-1.774518625	7.35E-11
ENSMUSG00000042109	Csdc2	0.5	73.5	-7.119761738	2.78E-09
ENSMUSG00000042684	Npl	1356	4379	-1.599666496	1.08E-13
ENSMUSG00000038732	Mboat1	30.75	401.75	-3.605603861	6.77E-12
ENSMUSG00000087651	1500009L16Rik	96	770	-2.922497801	1.02E-09
ENSMUSG00000046711	Hmga1	319	1909.25	-2.487323339	1.57E-14
ENSMUSG00000031490	Eif4ebp1	2508	5788.25	-1.115040182	8.25E-11
ENSMUSG00000029134	Plb1	71.25	493.25	-2.695106063	8.92E-12
ENSMUSG00000056214	Pard6g	29.25	187.5	-2.599339425	1.07E-08
ENSMUSG00000028645	Slc2a1	938.25	4548.25	-2.183713044	1.29E-14
ENSMUSG00000021094	Dhrs7	39.5	424.75	-3.35195818	1.4E-10
ENSMUSG00000039337	Tex19.2	47.75	318.25	-2.661189124	1.27E-10
ENSMUSG00000041062	Mslnl	0	71.75	-8.537234014	9.48E-12
ENSMUSG00000026917	Wdr5	1914.25	4455.5	-1.134280049	0.000000028
ENSMUSG00000021701	Plk2	118.5	1158.5	-3.198816107	3.69E-14
ENSMUSG00000034450	Gulo	657.75	3471.5	-2.324781064	1.35E-11
ENSMUSG00000022351	Sqle	256.75	1388.5	-2.344506501	5.83E-09
ENSMUSG00000031617	Tmem184c	95.5	440.75	-2.117035522	1.45E-11
ENSMUSG00000031604	Msmo1	277.25	869	-1.561778512	1.93E-11
ENSMUSG00000020097	Sgpl1	566.5	1419.25	-1.234647498	2.88E-09
ENSMUSG00000026888	Grb14	126.25	392.25	-1.546624708	2.05E-08
ENSMUSG00000021273	Fdft1	264	1222.25	-2.11883527	1.34E-23
ENSMUSG00000006386	Tek	0	45.5	-7.85958004	7.22E-10
ENSMUSG00000093930	Hmgcs1	548.25	1870.5	-1.678798455	1.32E-10
ENSMUSG00000066867	Oas1e	30.5	408.5	-3.630547932	7.51E-13
ENSMUSG00000040268	Plekha1	711	1815.25	-1.261116603	1.45E-11
ENSMUSG00000020883	Fbxl20	559.75	4255.25	-2.862982833	9.62E-12
ENSMUSG00000082292	Gm12250	0	36.25	-7.548548212	2.98E-08
ENSMUSG00000030605	Mfge8	765.75	2421.5	-1.584518899	4.74E-08
ENSMUSG00000019301	Hsd17b1	2.75	92.25	-5.007286322	4.24E-10
ENSMUSG00000019894	Slc6a15	7.75	126	-3.973374859	1.33E-08
ENSMUSG00000017144	Rnd3	434	2008	-2.134461913	8.55E-12

ENSMUSG00000046186	Cd109	25.5	314	-3.536163656	8E-11
ENSMUSG00000030268	Bcat1	1913	5037.25	-1.318957188	1.57E-08
ENSMUSG00000026791	Slc2a8	265.75	1617.75	-2.542516173	4.04E-11
ENSMUSG00000004359	Spic	473	3030.5	-2.624038855	7.36E-10
ENSMUSG00000038172	Ttc39b	140	481	-1.691767018	2.4E-12
ENSMUSG00000052316	Lrrc15	0	40.25	-7.69082042	6.24E-09
ENSMUSG00000029461	Fam168a	206.75	1065.25	-2.281423965	1.01E-09
ENSMUSG00000001670	Tat	562.25	3362	-2.506152676	3.72E-15
ENSMUSG00000018417	Myo1b	581.75	2133.5	-1.798125147	1.07E-10
